# Identification and validation of STEAP3 as a ferroptosis-related biomarker in heart failure

**DOI:** 10.3389/fcvm.2026.1751022

**Published:** 2026-06-01

**Authors:** Huijuan Chen, Lingqi Xu

**Affiliations:** 1 Department of Cardiology, The First Hospital of Changsha, Changsha, China; 2 School of Medicine, Yueyang Vocational Technical College, Yueyang, China

**Keywords:** aging, heart failure, machine learning, programmed cell death, steap3

## Abstract

**Background:**

Heart failure (HF) is a major health threat, with aging and programmed cell death (PCD) playing key roles. However, the link between aging-related PCD (aging-PCD) genes and HF remains unclear.

**Methods:**

We used ssGSEA and random forest to analyze PCD types in HF, identified aging-related PCDs via correlation analysis, and applied eight machine learning algorithms to develop a diagnostic model. SHAP and LIME were used to explain key features, and the relationship between the aging-PCD index and immune microenvironment was explored. Gene expression was verified by qRT-PCR, and the function of STEAP3 was further investigated in an H_2_O_2_-induced AC16 cell model. Ferroptosis-related changes were assessed by measuring reactive oxygen species (ROS), malondialdehyde (MDA), glutathione peroxidase (GSH-Px), and ferrous ion (Fe^2+^) levels.

**Results:**

Ferroptosis, autophagy, and necroptosis were strongly correlated with aging in HF. Eighteen differentially expressed aging-related PCD genes were identified. The LASSO model showed the best diagnostic performance. Significant differences in the immune microenvironment were observed between the aging-PCD index-high and index-low groups. A regulatory network of 15 key genes and 19 transcription factors was constructed. The qRT-PCR results validated the bioinformatics analysis. Functional experiments further suggested that STEAP3 may participate in ferroptosis-related injury in cardiomyocytes, potentially through glutathione metabolism (GPX4/SLC7A11 axis) and iron homeostasis.

**Conclusion:**

We identified aging-related PCD signatures in HF that may provide candidate biomarkers for further clinical validation.

## 1 Introduction

Heart failure (HF) is a clinical syndrome and end-stage manifestation of various cardiovascular diseases, characterized by a pathophysiological state in which the heart fails to meet metabolic demands due to impaired cardiac function under normal filling pressures (1). HF typically presents with symptoms such as tachycardia, dyspnea, pulmonary rales, pleural effusion, elevated jugular venous pressure, peripheral edema, and hepatomegaly (2). Major etiological factors include coronary artery disease, hypertension, cardiomyopathy, and valvular heart disease (1, 2). Despite advancements in treatment modalities, such as ACE inhibitors, beta-blockers, and diuretics, which improve symptoms and survival rates, the 5-year survival rate for HF remains below 50%, highlighting its poor prognosis. Current HF management encompasses pharmacological therapy, device-based interventions, and lifestyle modifications (3). While ACE inhibitors (ACEIs) and angiotensin receptor blockers (ARBs) form the cornerstone of pharmacological treatment, ACEIs may cause dry cough, leading to discontinuation in some patients (4). Device-based therapies, including cardiac resynchronization therapy (CRT) and implantable cardioverter-defibrillators (ICDs), are effective for select patients but are limited by high costs and procedural risks (5). These limitations underscore the urgent need for safer and more effective therapeutic strategies. The development of novel diagnostic models is crucial for early HF detection, prevention, and mechanistic exploration. Such models could provide a theoretical foundation for personalized treatment and improve patient outcomes.

Programmed cell death (PCD) is a genetically regulated process crucial for maintaining tissue homeostasis, eliminating damaged cells, and regulating development (6). Aging, characterized by the gradual decline of cellular and organ function, is a key biological process implicated in the pathogenesis of HF (7–9). Both PCD and aging play pivotal roles in HF progression. Apoptosis and necroptosis are primary mechanisms of cardiomyocyte loss in HF, while senescent cardiomyocytes contribute to myocardial fibrosis and cardiac remodeling through the aging-associated secretory phenotype (SASP), which releases pro-inflammatory factors and matrix metalloproteinases (MMPs) (10, 11). Additionally, aging-related endothelial dysfunction and vascular stiffening exacerbate HF pathology (12). Studies have shown that inhibiting PCD or clearing senescent cells can improve cardiac function and delay HF progression (13, 14). Investigating the interplay between aging and PCD in HF may uncover novel mechanistic insights and provide potential therapeutic targets for developing innovative treatment strategies. These findings highlight the significance of PCD and aging in HF pathogenesis and their potential as targets for therapeutic intervention.

In this study, we analyzed public transcriptomic datasets to identify aging-PCD related biomarkers in HF. We further explored their associated pathways, immune microenvironmental features, and the potential role of STEAP3 in ferroptosis-related injury, with the aim of providing new insights into HF pathogenesis and biomarker discovery.

## 2 Materials and methods

### 2.1 Collection of peripheral blood specimens

This study utilized peripheral blood samples obtained from 22 participants, comprising 11 patients with HF and 11 controls. Patients had heart failure due to ischemic cardiomyopathy. No subjects received mechanical support with left ventricular assist devices. Ages were comparable in subjects with HF (56 ± 11) and controls (55 ± 12; *P* = 0.49). All subjects were recruited from the Department of Cardiovascular Medicine at First Hospital of Changsha between June 2025 and July 2025. The study protocol was approved by the Ethics Committee in Clinical Research (ECCR) of First Hospital of Changsha (Approval No. 2025-8). All procedures were conducted in accordance with the ethical standards outlined in the Declaration of Helsinki.

### 2.2 Data source

HF transcriptome sequencing data were obtained from the Gene Expression Omnibus (GEO, https:// www. ncbi. nlm. nih. gov/ geo/query/acc.cgi) database. The GSE141910 dataset containing left ventricle samples from 195 HF patients and 161 controls was used as the training set, and the GSE57338 dataset containing left ventricle samples from 95 HF patients and 218 controls was used as the validation set. The training cohort (GSE141910) and validation cohort (GSE57338) were analyzed independently and were not merged for integrated expression analysis. Therefore, batch effect correction between the two datasets was not performed. Gene sets for apoptosis, pyroptosis, ferroptosis, autophagy, necroptosis, cuproptosis, parthanatos, entotic cell death, netotic cell death, lysosome-dependent cell death, alkaliptosis, oxeiptosis, and disulfidptosis were collected from previous literature (15). Meanwhile, a set of 307 aging genes was downloaded from the HAGR database (https:// www. genomics. senescence. info).

### 2.3 Identification of PCD pathways associated with aging

Based on the gene sets representing 13 PCD types, the ssGSEA algorithm was used to calculate the enrichment scores of 13 PCD for each sample in the training set. The random forest algorithm was utilized to rank differentially enriched PCD types in HF. Meanwhile, aging enrichment score for each sample in the training set was also calculated by ssGSEA, and aging-related PCD types were identified for subsequent analyses by Spearman correlation analysis (|cor| > 0.5, FDR < 0.05).

### 2.4 Identification and functional analysis of DEaging-PCD genes

The R package DESeq2 was used to identify genes with altered expression (DEGs) between HF and control samples in the training dataset using an adjusted *p*-value (adj p) < 0.05 and |log_2_ fold change (FC)| > 1 as criteria. DEGs and aging-related PCD (aging-PCD) genes were overlapped and defined as differentially expressed aging-related PCD genes (DEaging-PCD genes). Function enrichment analysis of DEaging-PCD was performed by ClueGO.

### 2.5 Construction and validation of diagnostic models

Machine learning provides a useful framework for constructing diagnostic models. Therefore, in this study, diagnostic models were constructed using logistic regression, LASSO, random forest, GradientBoosting, AdaBoost, XGBoost, GaussianNB and KNN methods in the training dataset and tested in the validation dataset. The optimal model was selected based on the accuracy, recall, precision, F1, kappa, Matthews correlation coefficient (MCC) and area under the curve (AUC) value of receiver operating characteristic (ROC) curves for eight machine learning algorithms in both training and validation cohorts. Additionally, SHAP and LIME algorithms were then used to further explain the contribution of model genes to the diagnostic model. To visualize the optimal model, a nomogram was constructed, and calibration and decision curve analyses were performed to evaluate its performance and potential clinical utility. Moreover, the diagnostic potential of each model gene was evaluated by ROC curves in both training and validation cohorts.

### 2.6 Gene set enrichment analysis (GSEA) of model genes

To explore the molecular mechanisms of model genes in regulating HF, GSEA was performed by downloading c2.cp.kegg.v2023.1.Hs.symbols from MSigDB database as reference gene sets. KEGG pathway with an adjusted *p* value < 0.05 were considered significantly enriched.

### 2.7 Effect of aging-PCD index on the immune microenvironment of HF

To understand the impact of the aging-PCD index on the immune microenvironment in HF, the aging-PCD index was calculated based on the expressions of model genes. HF samples were then classified into aging-PCD index^high^ and aging-PCD index^low^ groups. The differences in immune cell infiltration between these two groups were analyzed by CIBERSORT, XCELL, EPIC, QUANTISEQ, and MCPcounter algorithms. Meanwhile, the expressions of immunoactivating and immunosuppressing genes were also compared between two groups.

### 2.8 Construction of regulatory network

To predict transcription factors (TFs) regulating the expressions of model genes in HF, we first used the miRNet database (https:// www. mirnet. ca/) to retrieve TFs targeting model genes. Next, the predicted TFs was intersected with DEGs between HF and controls to obtain HF-associated TFs. Then HF-associated TFs and corresponding model genes were collected for constructing a TF-mRNA network.

### 2.9 Cell culture and cell transfection

Cells of the AC16 line were acquired from Procell (CL-0790, Wuhan, China). AC16 cells were cultured in Dulbecco's modified Eagle's medium/Nutrient Mixture F-12 DMEM/F-12 (Thermo Fisher Scientific, Shanghai, China) supplemented with 100 μg/mL streptomycin, 100 units/mL penicillin (1% P/S), and 10% foetal bovine serum (FBS) at 37 ℃ and 5% CO_2_. AC16 cell heart failure model was induced in a medium containing 200 μmol/L H_2_O_2_ for 24 h.

Small interfering RNA (siRNA) targeting STEAP3 and a negative control siRNA (si-NC) were obtained from Sangon Biotech (Shanghai, China). AC16 cells (1.0 × 10^6^ cells/well) were seeded into 6-well plates, each with 2 mL of complete medium. After incubation for 12 h, 100 nmol/L siRNA was transfected into the cells using Lipofectamine 3000 (Thermo Fisher Scientific, CA, USA) according to the instruction.

### 2.10 RT-qPCR

Total RNA of peripheral blood was isolated using Trizol Reagent (Beyotime), and RNAfast 200 purification kit (Fastagen) was used to extract total RNA of cells. The isolated RNA was then reverse transcribed into cDNA using ReverTra Ace qPCR RT Kit (TOYOBO). Real-time qPCR was performed using SYBR High-Sensitivity qPCR Supermix (Novoprotein) in ABI 7900HT (ABI). *β-Actin* was used as the internal control. The mRNA levels of each sample were evaluated using the 2^−*ΔΔ*CT^ method. The primers are listed in Table 1.

**Table 1:** Primer sequences for RT-qPCR and siRNA sequences used in this study. The table lists the primer sequences used for RT-qPCR analysis and the siRNA sequences used for STEAP3 knockdown experiments. All nucleotide sequences are presented in the 5’-3’ direction.

Primer	Sequence(5'-3’)
H-*β*-Actin-F	TGGACTTCGAGCAAGAGATG
H-β-Actin-R	GAAGGAAGGCTGGAAGAGTG
H-STEAP3-F	CCCTATGTGCAGGAAAGCCA
H-STEAP3-R	GGGCAAGTACACGAGTGACA
STEAP3-siRNA-495-F	GAGCAACCCUACAGAGCAATT
STEAP3-siRNA-495-R	UUGCUCUGUAGGGUUGCUCTT
STEAP3-siRNA-620-F	GCUUCAUGCCCGUGGACAUTT
STEAP3-siRNA-620-R	AUGUCCACGGGCAUGAAGCTT
STEAP3-siRNA-1216-F	UCGCUCAACUGGAGGGAGUTT
STEAP3-siRNA-1216-R	ACUCCCUCCAGUUGAGCGATT
Si-NC-F	UUCUCCGAACGUGUCACGUTT
Si-NC-R	ACGUGACACGUUCGGAGAATT
H-SLC7A11-F	CAGCTGTGGGCATAACTGTA
H-SLC7A11-R	ATTGCTGTGAGCTTGCAAAA
H-GPX4-F	GAGGCAAGACCGAAGTAAACTAC
H-GPX4-R	CCGAACTGGTTACACGGGAA

### 2.11 Measurement of Fe^2+^, glutathione peroxidase (GSH-Px), malondialdehyde (MDA) and ROS levels

For these assays, we harvested AC16 cardiomyocytes after treatment (H_2_O_2_ or/and transfection). According to the manufacturer's instructions, total cellular Fe^2+^ content was measured using a Ferrous Ion Content Assay Kit (Solarbio, Beijing, China), GSH-Px activity was measured using a Glutathione Peroxidase (GSH-PX) assay kit (Colorimetric method) (Jiancheng Bioengineering Institute, Nanjing, China), and MDA levels were measured using a Malondialdehyde (MDA) assay kit (TBA method) (Jiancheng Bioengineering Institute, Nanjing, China). For ROS detection, cells were incubated with 10 μM DCFH-DA (MCE, State of New Jersey, USA) for 30 min, and analyzed by flow cytometry.

### 2.12 Western blotting

For western blot analysis, whole-cell lysates were prepared. Total proteins were collected by centrifugation and then quantified by the BCA method (Beyotime). To run SDS-PAGE, equal amounts of total proteins were loaded onto 10% SDS gels. After electrophoresis, proteins were transferred onto nitrocellulose membranes and blocked in 5% non-fat milk at room temperature for 1 h. The membranes were washed and then incubated at 4℃ overnight with primary antibodies against GPX4 (Proteintech), SLC7A11 (Proteintech), STEAP3 (Proteintech), and GAPDH (DLMbiotech). After washing, HRP-conjugated secondary antibodies (Proteintech) were incubated with membranes in darkness for 0.5 h. ECL reagent (ABclonal) was added to the membranes to visualize the protein bands.

### 2.13 Statistical analysis

Data were analyzed by R and Python software. The Wilcoxon test was used to compare differences between two groups. FDR < 0.05, controlled by the Benjamini-Hochberg correction method, was considered statistically significant unless otherwise specified. All cell experiments were performed with three independent biological replicates, each including three technical replicates.

## 3 Results

### 3.1 Ferroptosis, autophagy, and necroptosis are correlated with aging in HF

**Figure 1:**
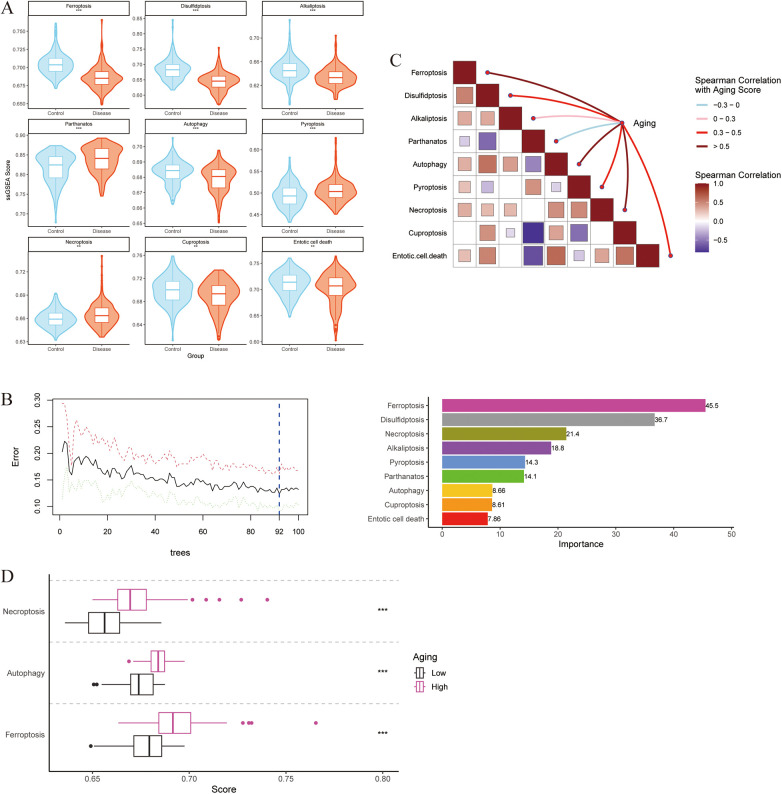
Identification of aginG,associated programmed cell death (PCD) patterns in heart failure (HF). (A) Comparison of ssGSEA enrichment score for differentially altered PCD pathways between control and HF samples in the training cohort. Violin plots show the distribution of pathway scores, and the embedded boxplots indicate the median and interquartile range. **(B)** Random forest analysis of differential PCD pathways. The left panel shows the relationship between the number of trees and model error rate, with the vertical dashed line indicating the selected optimal number of trees. The right panel presents the importance ranking of differential PCD pathways in distinguishing HF from control samples. **(C)** Spearman correlation analysis between the aging score and differential PCD pathway scores. The heatmap shows pairwise correlations among the differential PCD pathways, and the connecting lines indicate the correlations between each pathway and the aging score. Color and line width represent the strength and direction of the correlation. **(D)** Comparison of the ssGSEA scores of key aging-associated PCD pathways between the aging-score high and aging-score low HF subgroups. Boxplots show the median and interquartile range. Statistical significance is indicated as ***P < 0.001.

We observed that the enrichment scores of 9 PCDs were significantly different between HF and controls. Among them, the enrichment scores of ferroptosis, disulfidptosis, alkaliptosis, autophagy, cuproptosis, and entotic cell death were decreased, whereas the enrichment scores of parthanatos, pyroptosis, necroptosis enrichment scores were elevated in HF samples (Figure 1A). Random forest analysis identified ferroptosis, disulfidptosis and necroptosis as the top three PCD types contributing to HF (Figure 1B). Interestingly, we noticed that ferroptosis, autophagy, and necroptosis had strong correlations with aging (cor > 0.5, *p*-value < 0.05) (Figure 1C). To further support the close relationship between aging and those three PCDs, we divided HF patients into aging-score^high^ and aging-score^low^ groups, and those three PCDs were also significantly different between two aging-score groups (Figure 1D). Therefore, 522 genes involved in the ferroptosis, autophagy, and necroptosis were selected as aging-PCD genes for subsequent analyses.

### 3.2 A total of 18 DEaging-PCD genes were identified between HF and controls

**Figure 2:**
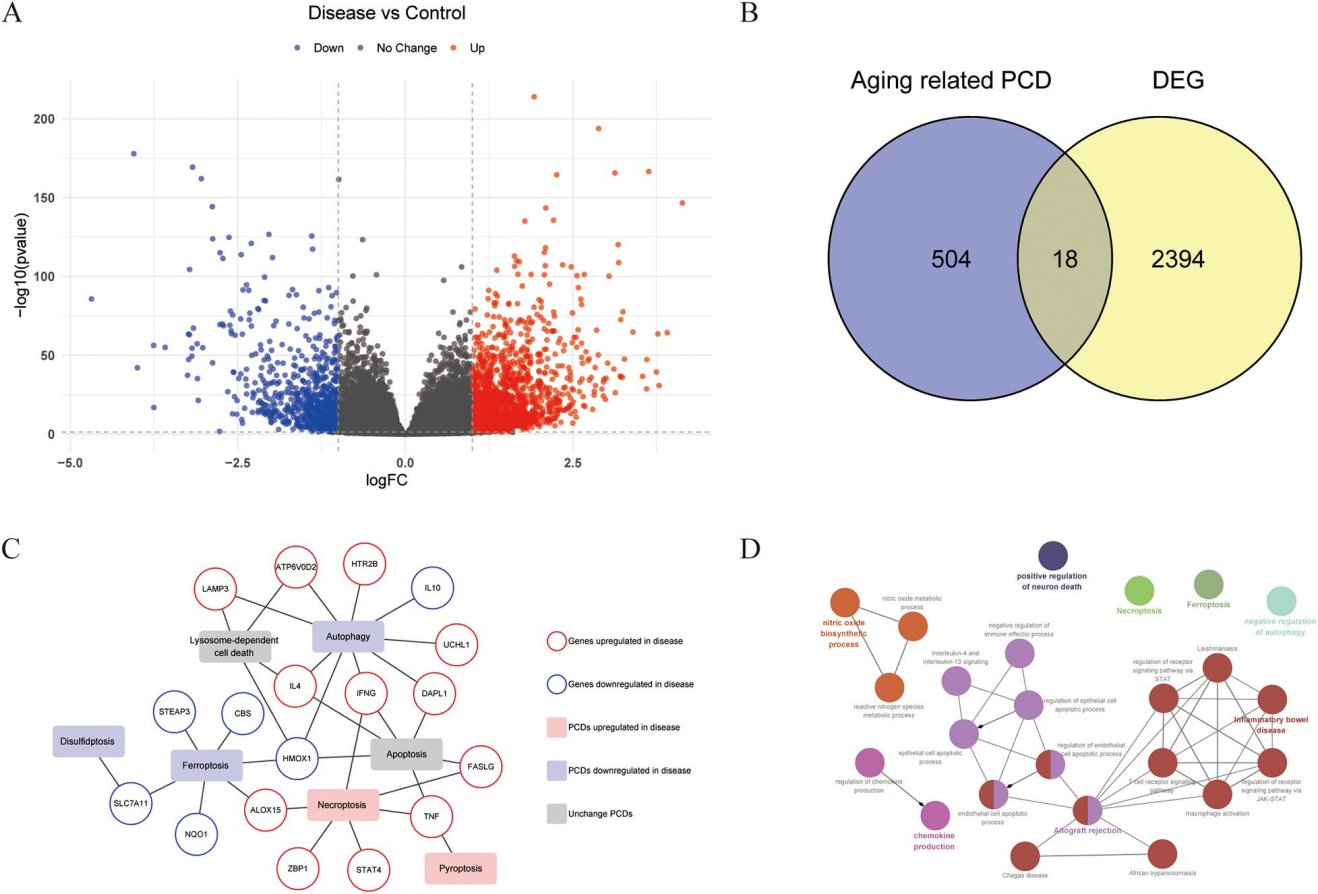
Identification and functional characterization of differentially expressed aging-related programmed cell death (PCD) genes in heart failure (HF). **(A)** Volvano plot showing differentially expressed genes (DEGs) between HF and control samples in the training cohort. Red dots indicate upregulated gens, blue dots indicate downregulated genes, and gray dots indicate genes without significant differential expression. **(B)** Venn diagram showing the overlap between DEGs and aging-related PCD genes. The intersecting genes were defined as differentially expressed aging-related PCD genes for subsequent analyses. **(C)** Network showing the associations between differentially expressed aging-related PCD genes and different PCD pathways. Genes are linked to their corresponding PCD categories. **(D)** Functional enrichment analysis of the differentially expressed aging-related PCD genes. The bubble plot shows significantly enriched biological processes and pathways. Bubble size represents the number of genes enriched in each term, and color indicates the significant level.

To obtain aging-PCD genes involved in HF, 2412 DEGs with 1703 genes upregulated and 709 genes downregulated in HF samples were screened **(**Figure 2A**)**. Next, 18 DEaging-PCD genes, including *LAMP3, ATP6V0D2, HTR2B, IL10, UCHL1, DAPL1, IFNG, IL4, STEAP3, CBS, HMOX1, SLC7A11, NQO1, ALOX15, TNF, FASLG, ZBP1* and *STAT4*, were obtained by intersecting 2412 DEGs with 522 aging-PCD genes **(**Figure 2B**)**. We found that those genes are not only involved in ferroptosis, autophagy, and necroptosis, but also participate in disulfidptosis and pyroptosis **(**Figure 2C**)**. Functional enrichment revealed that besides cell death, they were also associated with nitric oxide biosynthetic process, chemokine production, allograft rejection and inflammatory bowel disease **(**Figure 2D**)**. Collectively, these 18 DEaging-PCD genes may contribute to HF pathogenesis, primarily through regulation of PCD.

### 3.3 Diagnostic model for HF was developed and validated by machine learning

**Figure 3:**
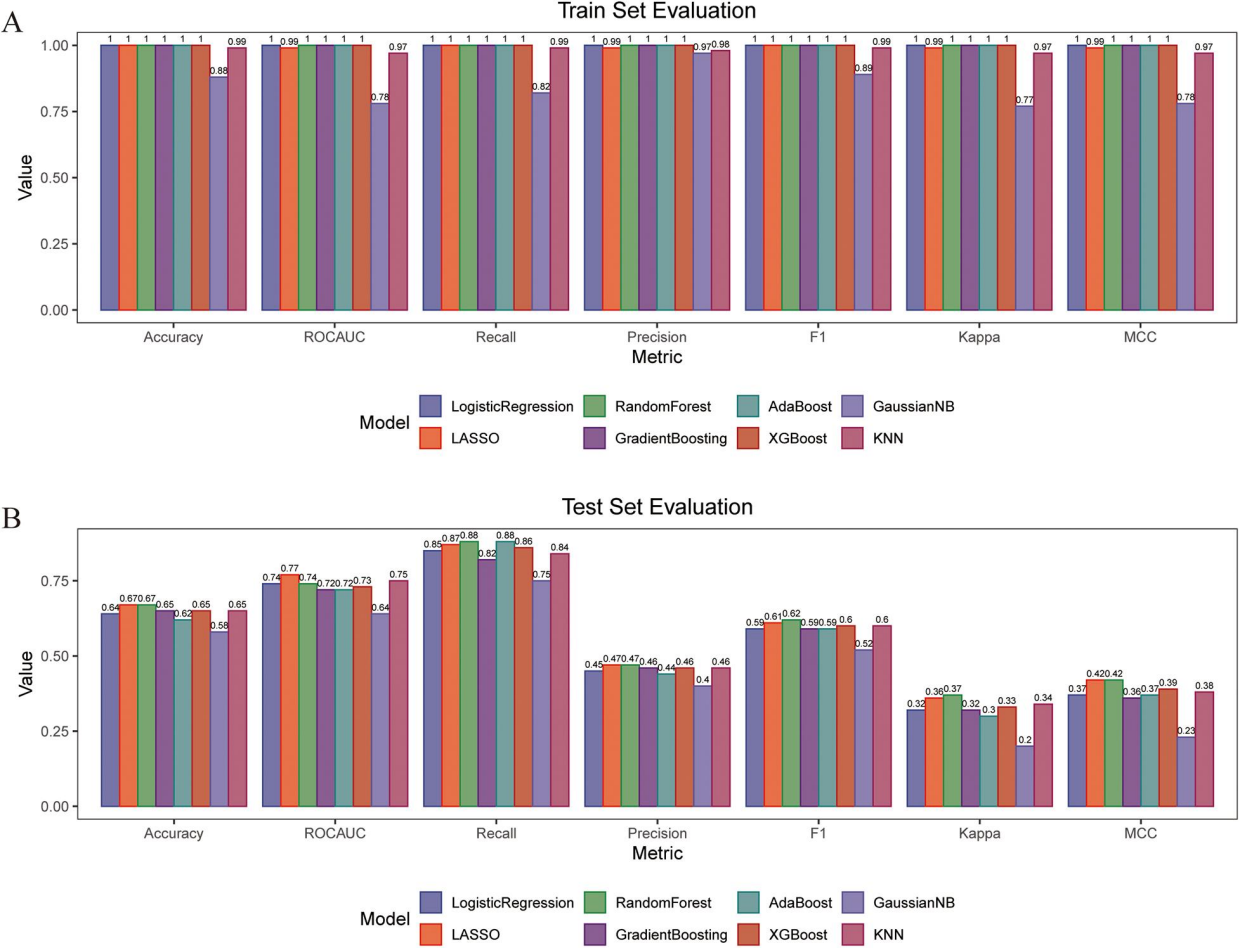
Comparison of machine learning model performance in the training and validation cohorts. **(A)** Performance metrics of eight machine learning algorithms in the training dataset. **(B)** Performance metrics of eight machine learning algorithms in the validation dataset. Evaluation metrics included accuracy, recall, precision, F1 score, kappa, MCC and AUC.

Subsequently, diagnostic models were constructed using logistic regression, LASSO, random forest, gradient boosting, AdaBoost, XGBoost, GaussianNB and KNN. As shown in Figure 3A, the performance of diagnostic models derived from logistic regression, LASSO, random forest, gradient boosting, and AdaBoost models were similar in the training cohort, as evidenced by multiple parameters, especially AUC values, but LASSO had the highest AUC value of 0.77 in the validation cohort **(**Figure 3B**)**. These results suggest that the LASSO model showed the most robust overall performance among the evaluated models in this study.

### 3.4 Interpretation of the optimal diagnostic model

**Figure 4:**
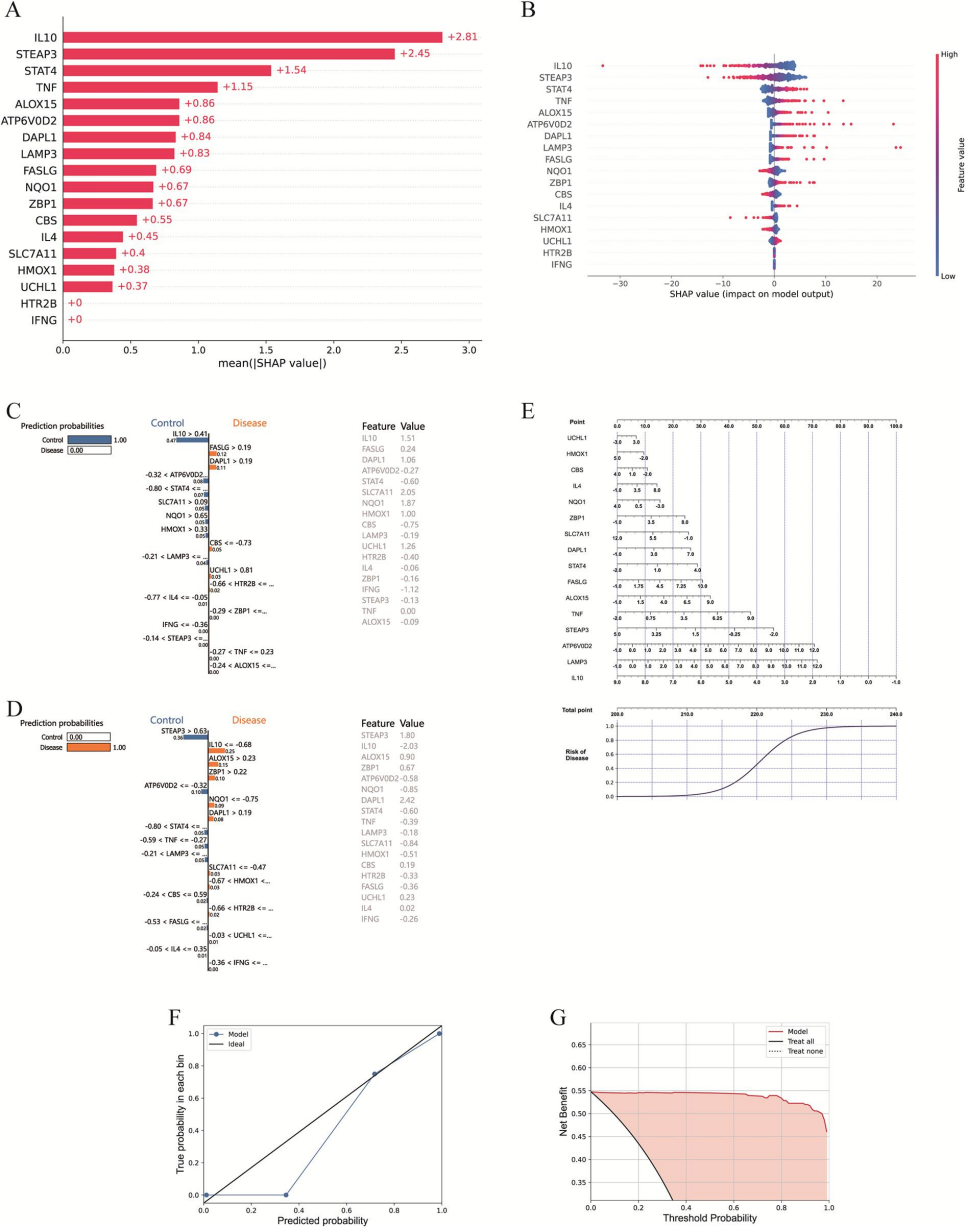
Interpretation and visualization of the optimal LASSO diagnostic model for heart failure (HF). (A) SHAP feature importance plot showing the relative contribution of model genes to the LASSO classifier. Features are ranked according to their mean absolute SHAP values. **(B)** SHAP summary plot illustrating the distribution of SHAP values for each model gene across all samples. Each dot represents one sample, and the color indicates the relative gene expression level. Positive SHAP values indicate a greater contribution to the prediction of HF. **(C)** Local interpretable model-agnostic explanations (LIME) plot for a representative control sample, showing the contribution of individual genes to the predicted class. **(D)** LIME plot for a representative HF sample, showing the contribution of individual genes to the predicted class. **(E)** Nomogram based on the LASSO model for estimating the probability of HF. Each predictor corresponds to a point value, and the total score is used to estimate the predicted risk. **(F)** Calibration curve of the nomogram showing the agreement between predicted and observed probabilities. The diagonal dashed line represents ideal calibration. **(G)** Decision curve analysis (DCA) of the nomogram showing the net clinical benefit across a range of threshold probabilities.

To interpret the LASSO model, SHAP analysis was first performed to quantify the contribution of each model gene. Genes were then ranked according to their SHAP values, showing that *IL10* and *STEAP3* contributed most strongly, followed by *STAT4, TNF* and other aging-related PCD genes **(**Figure 4A**)**. In the SHAP summary plot, each dot represents one sample, with red indicating higher gene expression and blue indicating lower gene expression. Positive SHAP values indicate a greater contribution to HF prediction, whereas negative SHAP values indicate a protective contribution. Specifically, the high IL10 expression with a negative SHAP value reduces the risk of HF, and the high STAT4 expression with a positive SHAP value leads to a higher risk of HF **(**Figure 4B**)**.

LIME analysis was further used to interpret representative sample-level predictions. For the representative control sample, the LASSO model predicted the control class with a probability of 1.00. IL10 was the dominant feature favoring the control classification, whereas FASLG, DAPL1, CBS, UCHL1 and HTR2B contributed toward the disease class (Figure 4C). For the representative HF sample, the model predicted the disease class with a probability of 1.00. Lower IL10 expression, together with higher ALOX15 and ZBP1 expression, lower NQO1 expression and higher DAPL1 expression favored the disease classification, whereas STEAP3 was the strongest feature supporting the control class (Figure 4D). A nomogram was then constructed to visualize the model, and calibration and decision curve analyses suggested good model performance (Figures 4E–G).

### 3.5 Characterization of the aging-PCD model genes in HF

**Figure 5:**
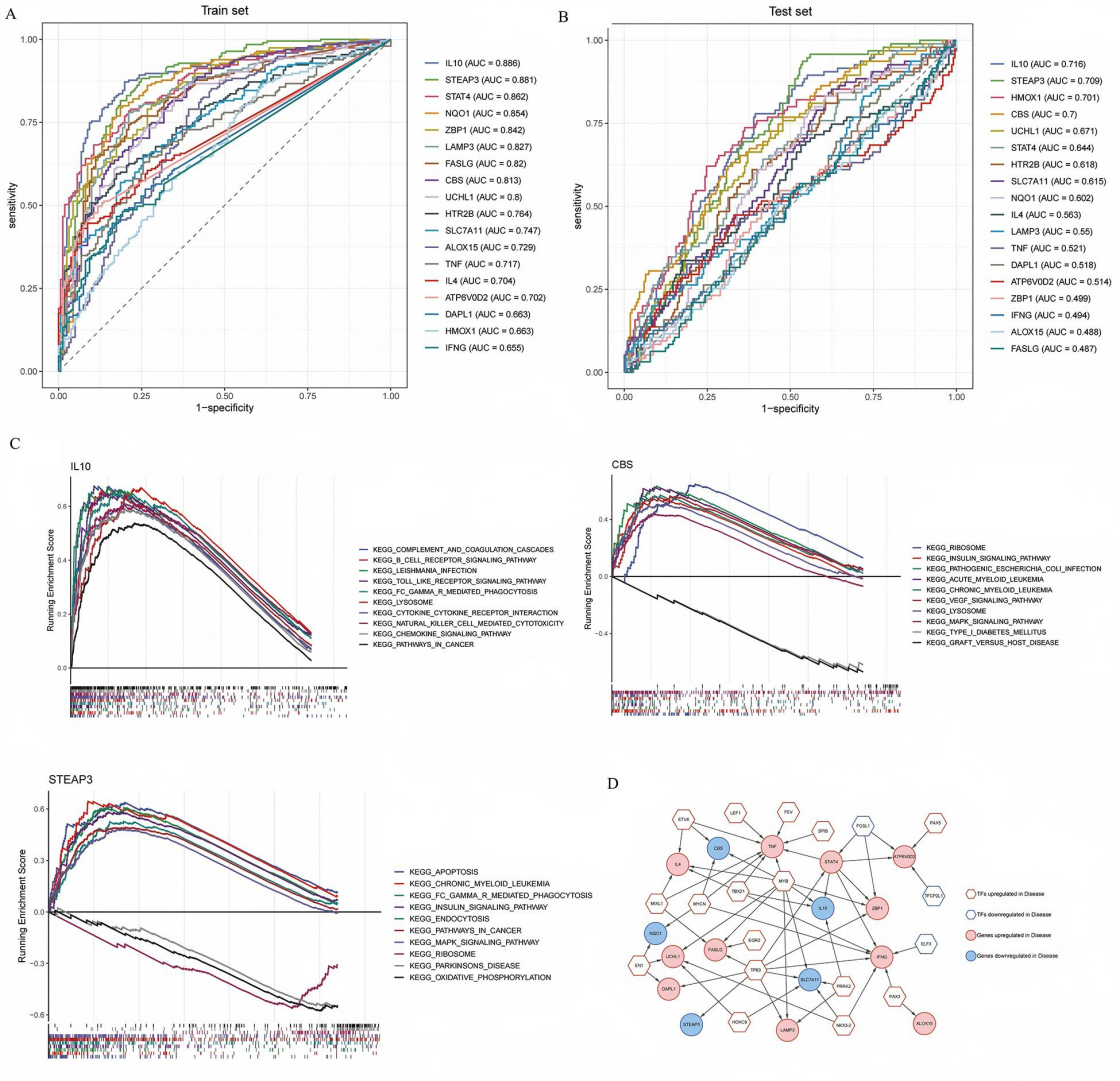
Characterization of model genes in heart failure (HF). (A) Receiver operating characteristics (ROC) curves showing the diagnostic performance of individual model genes in the training cohort. The area under the curve (AUC) for each gene is indicated in the legend. **(B)** ROC curves showing the diagnostic performance of individual model genes in the validation cohort. The AUC for each gene is indicated in the legend. **(C)** Gene set enrichment analysis (GSEA) for representative model genes. Enrichment plots show significantly associated KEGG pathways for IL10, CBS and STEAP3. **(D)** Transcription factor (TF)-mRNA regulatory network of model genes in HF. Circular nodes represent model genes, hexagonal nodes represent TFs, red indicates upregulation in HF, and blue indicates downregulation in HF.

The diagnostic efficacy of individual model genes in HF was also evaluated using ROC curve analysis. The ROC curves in training and validation datasets revealed that *IL10, STEAP3,* and *CBS* genes exhibited promising diagnostic potential. Specifically, in the GSE141910 dataset, the AUC value for *IL10, STEAP3, CBS* reached 0.886, 0.881 and 0.813, respectively **(**Figure 5A**)**. In the GSE57338 dataset, the AUC values were 0.716 for *IL10*, 0.709 for *STEAP3*, and 0.700 for *CBS* **(**Figure 5B**)**. Furthermore, we applied GSEA to analyze the function of each model gene. GSEA suggested that *IL10* and *STEAP3* were associated with apoptosis, *IL10* and *CBS* with lysosome pathways; and *STEAP3* and *CBS* with insulin signaling pathway, ribosome-associated processes, and endocytosis. In addition, IL10 was associated with cytokine-cytokine receptor interaction and natural killer cell mediated cytotoxicity, *STEAP3* with oxidative phosphorylation, and *CBS* with MAPK and VEGF signalling pathway (Figure 5C). Additional GSEA results are shown in Supplementary Figures S1, S2.

In addition, a TF-mRNA regulatory network comprising 15 model genes and 19 TFs was constructed, with red indicating upregulation in HF and blue indicating downregulation (Figure 5D). Within this network, *TNF* showed the broadest predicted TF regulation, including *ETV4, LEF1, FEV, SPIB, MYB, TBX21, MYCN* and *MIXL*. *IL10* was predicted to be regulated by *MYB, TBX21* and *PRRX2*, whereas CBS was predicted to be regulated by *ETV4, MYCN* and *MYB*.

### 3.6 The aging-PCD index was closely associated with the immune microenvironment in HF

**Figure 6:**
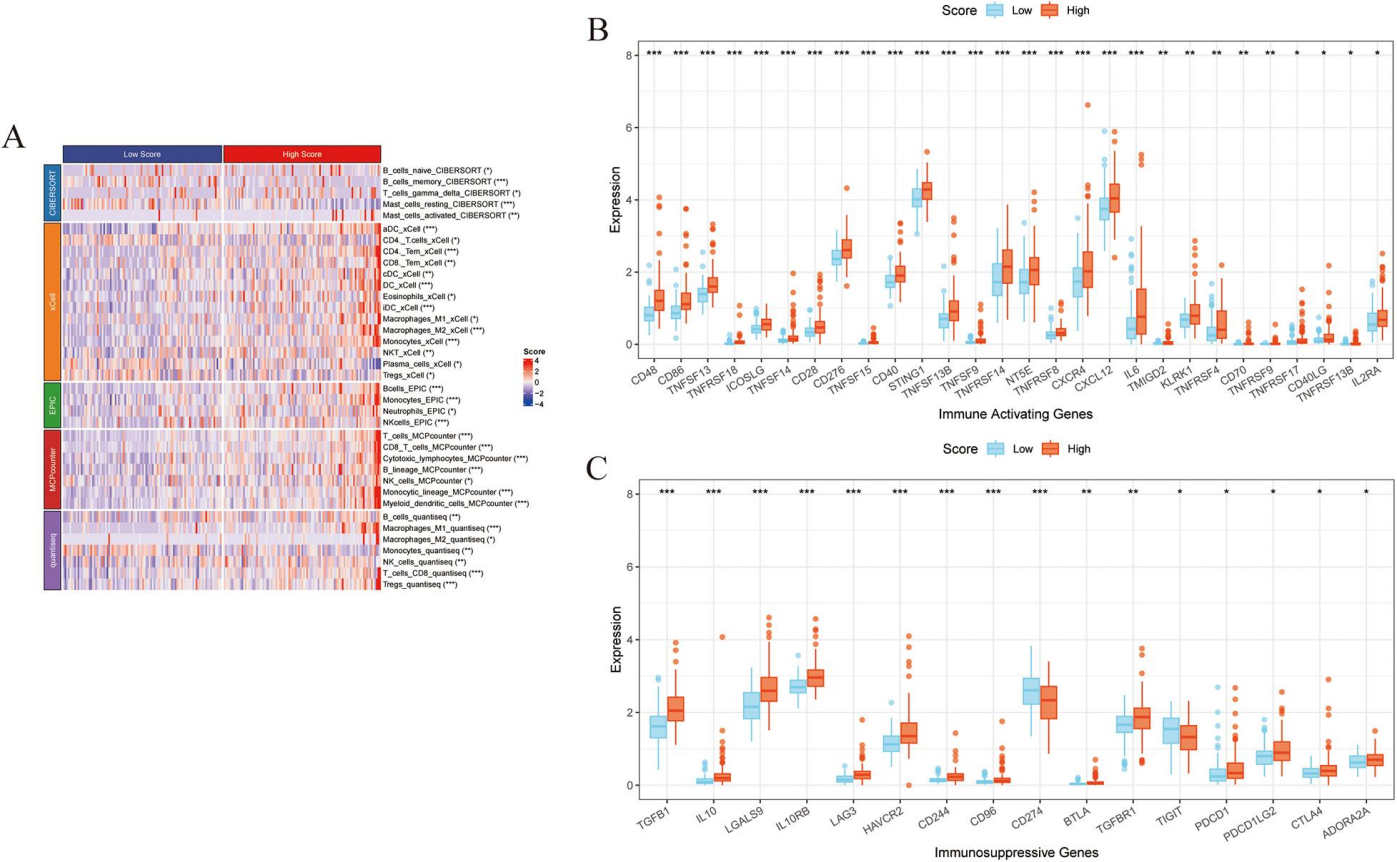
Association between the aginG–PCD index and the immune microenvironment in heart failure (HF). (A) Heatmap showing differences in immune cell infiltration between the aging-PCD index high and aging-PCD index low HF subgroups, as estimated by multiple immune deconvolution algorithms, including CIBERSORT, xCell, EPIC, quanTIseq and MCP-counter. **(B)** Boxplot showing differentially expressed immune-activating genes between the aging-PCD index high and aging-PCD index low HF subgroups. **(C)** Boxplot showing differentially expressed immune-suppressive genes between the aging-PCD index high and aging-PCD index low HF subgroups.

To further characterize the relationship between the aging-PCD index and the immune microenvironment in HF, we compared immune infiltration patterns between aging-PCD index^high^ and aging-PCD index^low^ subgroups. Significant differences were observed in CD4 T cells, CD8 T cells, macrophages, B cells, and natural killer (NK) cells (Figure 6A). Additionally, 28 immune-activating genes, such as *CD48, CD86, TNFSF13, STING1, IL6*, and 16 immune-suppressing genes, such as *TGFB1, IL10, LAG3,* and *CD27*4, exhibited significant differences between the aging-PCD index^high^ and aging-PCD index^low^ groups, further indicating the different immune microenvironment between two aging-PCD groups (Figures 6B,C).

### 3.7 STEAP3 was associated with ferroptosis-related injury of cardiomyocytes

**Figure 7:**
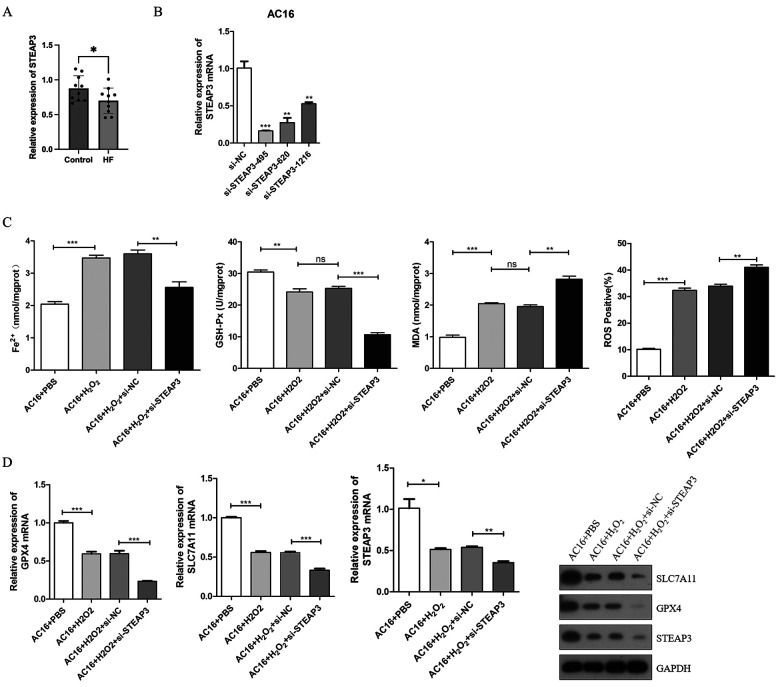
Preliminary validation of STEAP3 expression and its association with ferroptosis-related injury in H_2_O_2_-treated AC16 cells. (A) Relative expression of STEAP3 in peripheral blood samples from control subjects and patients with HF. (B) Knockdown efficiency of three siRNA targeting STEAP3 in AC16 cells as determined by RT-qPCR. (C) Effects of STEAP3 silencing on intracellular Fe^2+^, glutathione peroxidase (GSH-Px) activity, malondialdehyde (MDA) level and the percentage of ROS-positive cells. (D) The mRNA and protein expressions of GPX4, SLC7A11 and STEAP3 in AC16 cells. Relative mRNA expression was measured by RT-qPCR and protein levels were detected by western blotting using GAPDH as the loading control. Data are presented as mean ± SD. Statistical significance is indicated as ^ns^P >0.05, *P < 0.05, **P < 0.01, ***P < 0.001.

Based on the AUC values from the training and validation sets, genes with an AUC greater than 0.7 (*IL10 and STEAP3*) were selected for further investigation. However, given the extensive existing literature on *IL10* and the relatively unclear role of *STEAP3* in HF, we focused on *STEAP3* for subsequent studies. Initially, a significant downregulation of *STEAP3* was observed in HF (Figure 7A), suggesting its potential involvement in HF pathogenesis. To explore its functional role, *STEAP3* was silenced in AC16 cardiomyocytes using three distinct siRNAs (*STEAP3*-siRNA-495, *STEAP3*-siRNA-620 and *STEAP3*-siRNA-1216). Among these, *STEAP3*-siRNA-495 demonstrated higher interference efficiency and was therefore selected for further experiments. (Figure 7B). Functionally, STEAP3 knockdown increased ferroptosis-related oxidative stress, as evidenced by decreased GSH-Px activity and increased MDA and ROS levels compared with negative-control transfection. In addition, the concentration of Fe^2+^ was reduced after STEAP3 silencing (Figure 7C). Consistently, ferroptosis-related effectors were repressed at both the mRNA and protein levels, with *GPX4* and *SLC7A11* decreased following *STEAP3* knockdown (Figure 7D). Collectively, these data suggest that *STEAP3* may be involved in ferroptosis-related injury in cardiomyocytes, potentially through glutathione metabolism (*GPX4*/*SLC7A11* axis) and iron homeostasis.

## 4 Discussion

HF, the terminal stage of cardiovascular disease, is characterized by high mortality and poor prognosis, and can be triggered by a variety of clinical factors (16). Recent studies have demonstrated that the risk of HF increases with age, cellular senescence, and cell death, highlighting the critical roles of these processes in HF pathogenesis (17). Building on these insights, this study developed an optimized diagnostic model for HF based on aging-associated PCD-related genes. Furthermore, we investigated the pathways and regulatory networks associated with these model genes. These findings provide new insight into aging-related PCD signatures in HF and highlight candidate biomarkers for further investigation.

Among the various forms of PCD implicated in the pathogenesis of HF, ferroptosis, autophagy, and necroptosis exhibit significant differences between disease and control groups. Their PCD scores show the strongest correlation with aging scores, underscoring their critical roles in HF progression. Ferroptosis, an iron-dependent form of cell death driven by lipid peroxidation (18, 19), has emerged as a key contributor to myocardial injury. Autophagy, a cellular degradation mechanism responsible for recycling damaged organelles and protein aggregates, plays a dual role in HF. Moderate activation of autophagy protects cardiomyocytes by mitigating oxidative stress and apoptosis through the clearance of dysfunctional mitochondria and protein aggregates (20). However, excessive autophagy can lead to detrimental degradation of cellular components, exacerbating cell death and HF progression (21). Experimental evidence from HF rat models has consistently demonstrated concurrent upregulation of autophagy and ferroptosis (22, 23). Furthermore, *in vitro* studies have identified TLR4 as a critical regulator mediating cardiomyocyte loss through the modulation of both autophagy and ferroptosis during HF progression (24). These findings suggest that cardiomyocytes in HF are subjected to oxidative stress, resulting in disrupted iron homeostasis and elevated lipid peroxidation, which collectively trigger ferroptosis. Simultaneously, autophagy may influence HF pathogenesis by regulating oxidative stress and apoptotic pathways. In addition to these mechanisms, necroptosis has been shown to contribute significantly to HF development through its roles in mediating cardiomyocyte death, inflammatory responses, and cardiac remodeling (25). Pharmacological or genetic modulation of necroptosis levels may therefore represent a promising therapeutic strategy to improve cardiac function and delay HF progression. Further investigation into the regulatory mechanisms of necroptosis could provide novel insights for HF treatment.

Using eight machine learning algorithms, we identified a 15-gene signature associated with HF and found that the LASSO model showed the most robust overall performance. Among the model genes, IL10, STEAP3 and STAT4 merged as prominent contributors and may be linked to inflammation, oxidative stress and PCD. Notably, IL10 and STEAP3 demonstrated superior diagnostic potential, with individual AUC values exceeding 0.7, highlighting their robust predictive value for HF.

*IL10*, an anti-inflammatory cytokine, plays a pivotal role in attenuating inflammatory responses by suppressing pro-inflammatory signaling pathways and modulating immune cell functions (26). Experimental evidence indicates that IL-10 mitigates myocardial inflammation and fibrosis by inhibiting the pro-inflammatory activities of macrophages and T cells, thereby reducing the release of cytokines such as *TNF-*α and *IL-6* (27). *STAT4*, a critical transcription factor in the JAK-STAT signaling pathway, primarily mediates IL-12 and IFN-*γ* signaling to promote Th1 cell differentiation and pro-inflammatory responses (28). However, its excessive activation has been linked to chronic inflammation and myocardial injury, contributing to cardiomyocyte apoptosis and cardiac dysfunction in HF patients (29, 30).

*STEAP3*, a metalloreductase involved in iron metabolism and oxidative stress responses, regulates intracellular iron homeostasis and influences oxidative stress levels (31). In HF, iron overload and oxidative stress are known to exacerbate cardiomyocyte damage (19), suggesting that *STEAP3* may regulate iron-dependent cell death (ferroptosis), thereby aggravating HF progression. Our qPCR analysis revealed a significant downregulation of *STEAP3* in HF patients compared to controls, suggesting its potential involvement in HF pathogenesis. In AC16 cells, *STEAP3* knockdown aggravated ferroptotic stress that GSH-Px activity decreased, while MDA and the percentage of ROS-positive cells increased compared with negative-control transfection. However, we observed that total cellular Fe^2+^ was reduced in our assay. STEAP3 is an endosomal ferrireductase that reduces Fe^3+^ to Fe^2+^ during transferrin-dependent iron uptake (32). Therefore, knockdown of STEAP3 may directly reduce the measurable intracellular ferrous ion pool by impairing ferric-to-ferrous iron conversion and altering intracellular iron trafficking. At the same time, ferroptotic susceptibility is not determined solely by total cellular Fe2 + abundance, but also by antioxidant defense capacity and the subcellular distribution of redox-active iron (33, 34). In our study, STEAP3 knockdown was accompanied by decreased GSH-Px activity, increased MDA, increased ROS and reduced GPX4 and SLC7A11 expressions, which together support enhanced ferroptosis-related oxidative stress and impaired anti-ferroptotic defense. These findings suggest that STEAP3 may participate in ferroptosis-related oxidative stress and may exert a protective role in cardiomyocytes. However, because the current evidence is mainly derived from knockdown-based *in vitro* experiments, the precise mechanistic role of STEAP3 in ferroptosis regulation and Fe^2+^ level requires further investigation by overexpression, rescue and pharmacological validation studies.

GSEA revealed that *IL10, STEAP3* and *CBS* play critical roles in HF by modulating key biological processes, including apoptosis, lysosomal function, oxidative phosphorylation, and signaling pathways. Apoptosis, a central pathological mechanism in HF, is closely associated with the decline in cardiac function, as evidenced by increased cardiomyocyte apoptosis in HF models (10). Furthermore, apoptosis is intricately linked to other pathological processes such as myocardial fibrosis, oxidative stress, and inflammatory responses, which collectively drive HF progression (35–37). Lysosomal dysfunction in HF is primarily characterized by impaired autophagy and ferroptosis. Mitochondrial dysfunction, which disrupts NAD + levels, leads to lysosomal acidification and autophagy impairment. Additionally, mitochondrial dysfunction-induced lysosomal damage triggers ferroptosis, a process marked by iron accumulation and lipid peroxidation within lysosomes, further exacerbating HF pathology (38, 39). Oxidative phosphorylation, a core process in myocardial energy metabolism, is significantly compromised in HF. Mitochondrial dysfunction impairs oxidative phosphorylation, reducing ATP production and thereby diminishing energy supply and contractile function in cardiomyocytes (40, 41). Moreover, the decline in NAD + levels in HF reduces *SIRT3* activity, which adversely affects complex I function and further disrupts oxidative phosphorylation (42). *IL10*, *STEAP3*, and *CBS* regulate HF through distinct yet interconnected mechanisms involving apoptosis, lysosomal function, and oxidative phosphorylation. These findings highlight the complex interplay of cellular processes in HF pathogenesis and provide potential targets for therapeutic intervention.

In this study, the stratification of patients based on aging-PCD indices revealed significant differences in immune cell abundance and immune-related gene expression, suggesting distinct immune microenvironments between high and low aging-PCD groups. Research indicates that T cell function declines during aging, characterized by an imbalance in the CD4+/CD8 + ratio and T cell exhaustion (43). In HF patients, dysregulated T cell function may exacerbate inflammatory responses and tissue damage. Among immune activation genes, *IL6*, a central mediator of inflammaging, is associated with HF progression and poor prognosis due to its elevated expression (44). Additionally, the STING1 pathway, activated during aging, may aggravate HF by promoting inflammation and cell death (45).

Immune suppression genes such as *TGFB1, IL10, LAG3* and *CD274* are linked to immune suppression and immune evasion (46). *TGFB1* and *IL10*, key immunosuppressive factors, may promote tissue fibrosis by inhibiting T cell function (47). *LAG3* and *CD274*, markers of T cell exhaustion, are associated with impaired anti-tumor and anti-infection immune responses when overexpressed (48). These findings suggested that personalized immunomodulatory strategies may be necessary for HF patients based on their aging-PCD profiles. Future studies should further explore the prognostic and therapeutic potential of these immune markers in HF management.

This study leveraged transcriptomic data from public databases and bioinformatics approaches to investigate the diagnostic potential of aging-associated PCD genes in HF, culminating in the development of a diagnostic model. Although our study provides valuable insights, several limitations should be acknowledged. First, the diagnostic model was developed from left ventricular tissue transcriptomic data, whereas the experimental validation was performed in peripheral blood samples. Because these two sample types are biologically distinct, the present study does not yet support the direct application of this model to peripheral blood-based clinical diagnosis. Further studies should include independent peripheral blood cohorts to further validate the translational feasibility of the model and its core biomarkers. Second, the clinical sample size was relatively small and was mainly used for preliminary validation of STEAP3 expression. Larger-scale and multicenter cohorts will be required in future studies to further confirm its clinical relevance and generalizability. Third, the mechanistic evidence supporting STEAP3 as a ferroptosis regulator is still preliminary, as the current functional validation was mainly based on knockdown experiments and ferroptosis-related indicators. More direct evidence, including overexpression assays, rescue experiments, and validation with classical ferroptosis inhibitors or inducers, is required to further define the specific role of STEAP3 in ferroptosis regulation. In addition, the functional experiments in this study were limited to an H_2_O_2_-induced AC16 cell model, and no *in vivo* validation was performed. Therefore, the mechanistic role of STEAP3 in heart failure requires further confirmation in animal models. In future studies, we will validate our findings in established heart failure animal models, such as TAC- or isoproterenol-induced models, combined with cardiac function assessment and ferroptosis-related analyses, to further strengthen the robustness and translational relevance of our conclusions.

## 5 Conclusions

In summary, we identified aging-related PCD signatures in HF and developed a tissue-based molecular diagnostic model with potential biomarker value. Among the identified genes, STEAP3 was downregulated in HF and may participate in ferroptosis-related injury in cardiomyocytes through glutathione metabolism and iron homeostasis.

## Data Availability

The original contributions presented in the study are included in the article/Supplementary Material, further inquiries can be directed to the corresponding author.

## Supplementary material

Figure S1 GSEA for the ATP6V0D2, ALOX15, DAPL1, FASLG, HMOX1, HTR2B, IFNG, IL4, LAMP3, NQO1, SLC7A11 and STAT4.

Figure S2 GSEA for the *TNF*, *UCHL1* and *ZBP1.*
