# Supplementary figures and images for: Identification and validation of STEAP3 as a ferroptosis-related biomarker in heart failure
Source: Front Cardiovasc Med. 2026 Jun 1;13:1751022. doi: 10.3389/fcvm.2026.1751022 (PMC13265469; doi:10.3389/fcvm.2026.1751022)

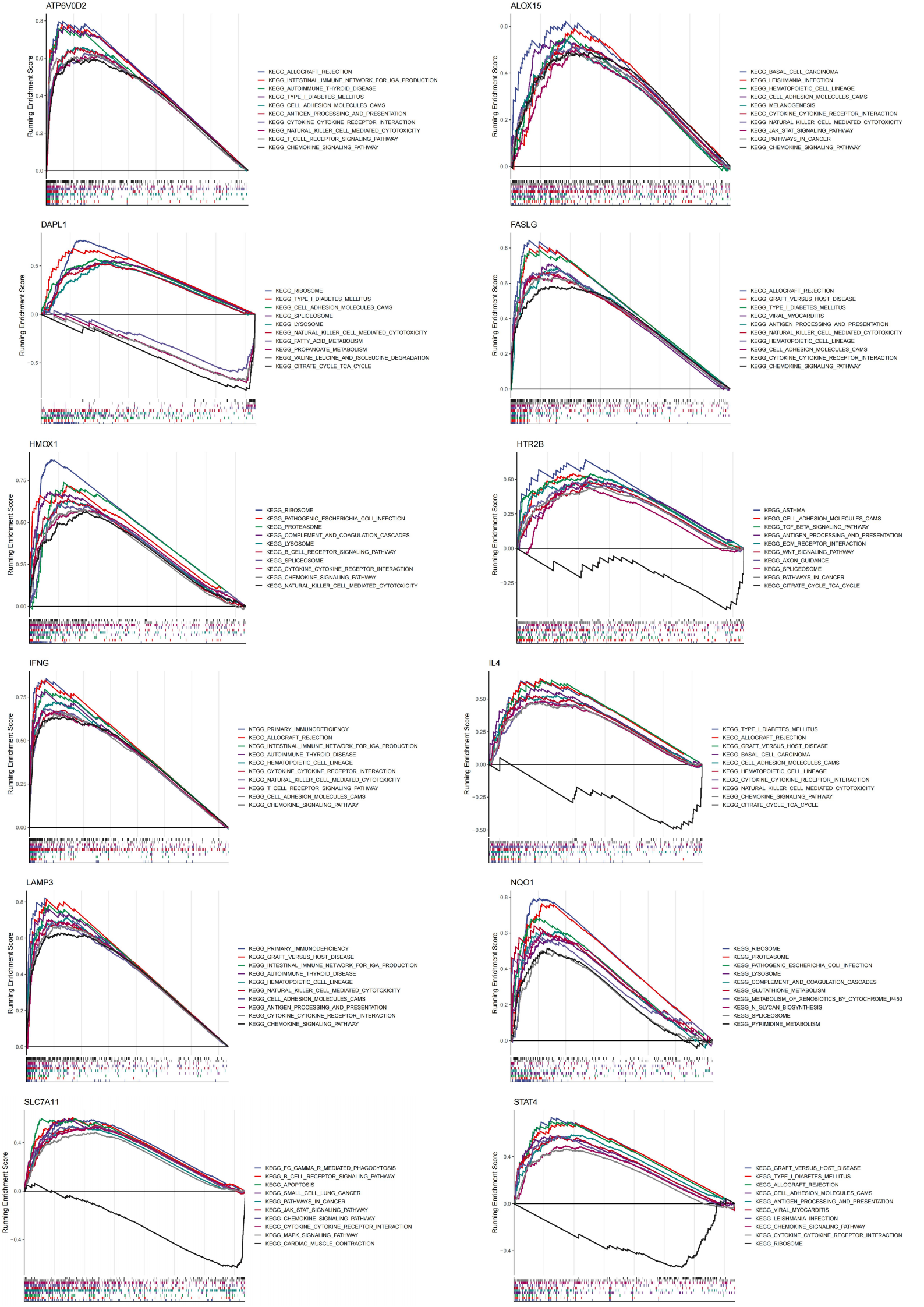

Supplement: Figure S1 — GSEA for the ATP6V0D2, ALOX15, DAPL1, FASLG, HMOX1, HTR2B, IFNG, IL4, LAMP3, NQO1, SLC7A11 and STAT4. [file Image1.tif]

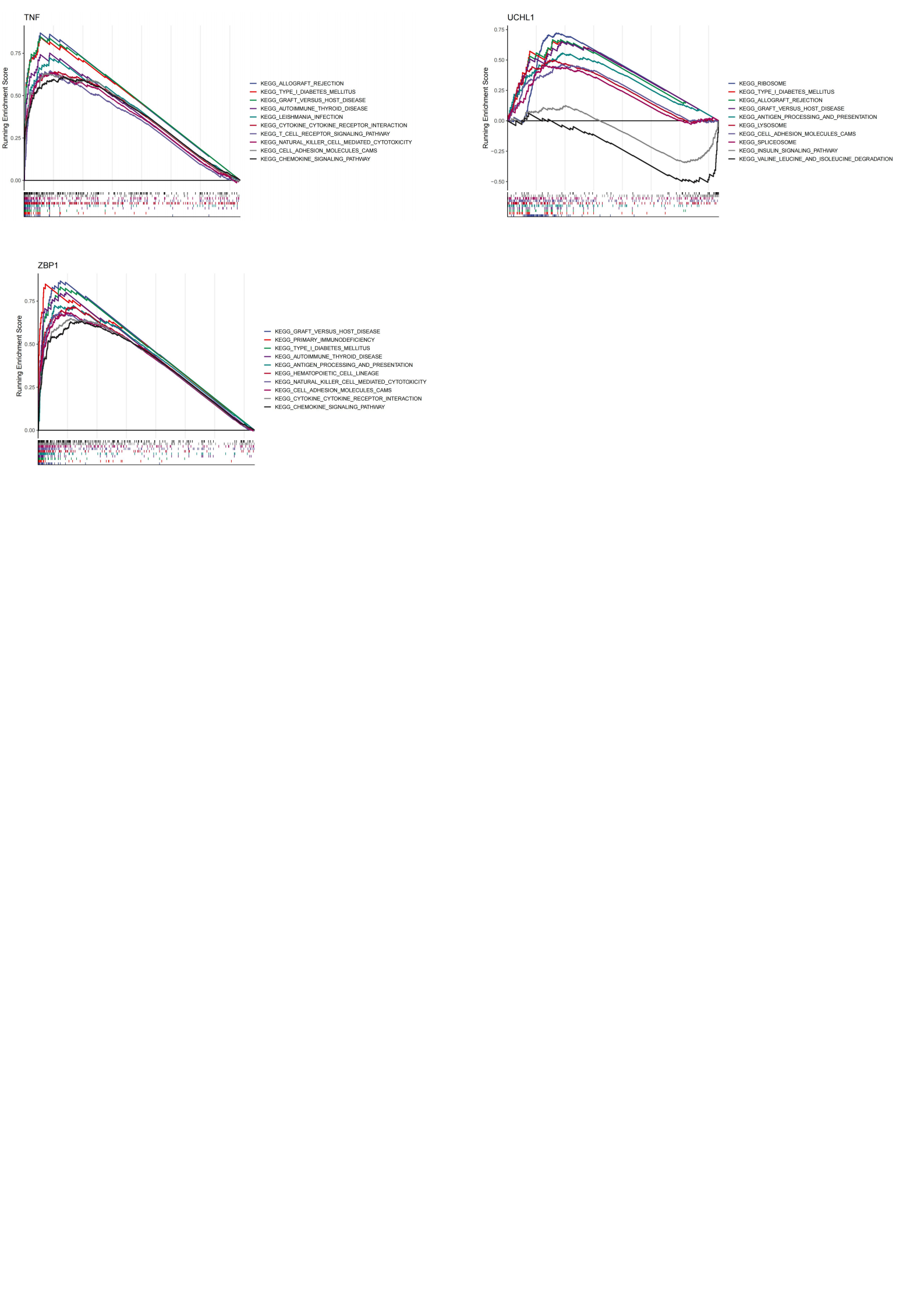

Supplement: Figure S2 — GSEA for the TNF, UCHL1 and ZBP1. [file Image2.tif]
